# Junctional Adhesion Molecules: Potential Proteins in Atherosclerosis

**DOI:** 10.3389/fcvm.2022.888818

**Published:** 2022-07-07

**Authors:** Junqi Wang, Xiaoping Chen

**Affiliations:** ^1^Department of Clinical Pharmacology, Xiangya Hospital, Central South University, Changsha, China; ^2^Hunan Key Laboratory of Pharmacogenetics, Institute of Clinical Pharmacology, Central South University, Changsha, China; ^3^National Clinical Research Center for Geriatric Disorders, Xiangya Hospital, Central South University, Changsha, China

**Keywords:** junctional adhesion molecules, atherosclerosis, inflammation, thrombosis, transendothelial migration

## Abstract

Junctional adhesion molecules (JAMs) are cell-cell adhesion molecules of the immunoglobulin superfamily and are involved in the regulation of diverse atherosclerosis-related processes such as endothelial barrier maintenance, leucocytes transendothelial migration, and angiogenesis. To combine and further broaden related results, this review concluded the recent progress in the roles of JAMs and predicted future studies of JAMs in the development of atherosclerosis.

## Introduction

A junctional adhesion molecule (JAM) is a member of the immunoglobulin superfamily (IgSF) (1–3), a large superfamily of cell surface and soluble proteins that are involved in the recognition, binding, or adhesion of cells. The membrane-located JAMs are immunoglobulin-like single-span transmembrane molecules expressed by leukocytes, platelets, epithelial and endothelial cells, localizing to cell-cell contacts and specifically enriched at tight junctions (2, 3).

Atherosclerosis (AS) plaques comprise lipids, fibers, and immune cells in the intima of large and medium-sized arteries, and immunological components are indispensable in both the initiation and the chronicity of the lesions (4). Several immune activities such as platelet aggregation and adhesion and the transendothelial migration (TEM) of monocytes and neutrophils (4, 5) are increasingly recognized as the leading cause of atherosclerosis. JAMs present more and more association with AS, as considerable molecules in vascular inflammation.

Thus, we briefly reviewed the classification, structure, primary ligands and receptors, and main physiological functions of JAMs, and summarized and speculated potential roles of JAMs in AS based on reported articles.

## Junctional Adhesion Molecules Overview

### Classification and Structure

Current studies have focused on four major JAM molecules: JAM1, JAM2, JAM3, and JAM4 (6), also known as JAM-A, JAM-B, JAM-C, and JAM4 (7). Moreover, some other proteins are closely related to JAMs, including JAML (JAM-like) (8), CLMP [CAR (coxsackie and adenovirus receptor)-like membrane protein] (9), CAR (10), and ESAM (endothelial cell adhesion molecule) (11). Martìn-Padura et al. first reported JAMs as a new member of the immunoglobulin family concentrating endothelial and epithelial junctions and identified JAM1 (12). Further studies cloned JAM2 and JAM3 and identified them as counter-receptor (13–17). The shedding of JAM-A produces soluble JAM-A (sJAM-A). For instance, transmembrane-JAM-A was shed to generate proinflammatory sJAM-A and JAM-A-bearing microparticles when platelets were activated.

**FIGURE 1:**
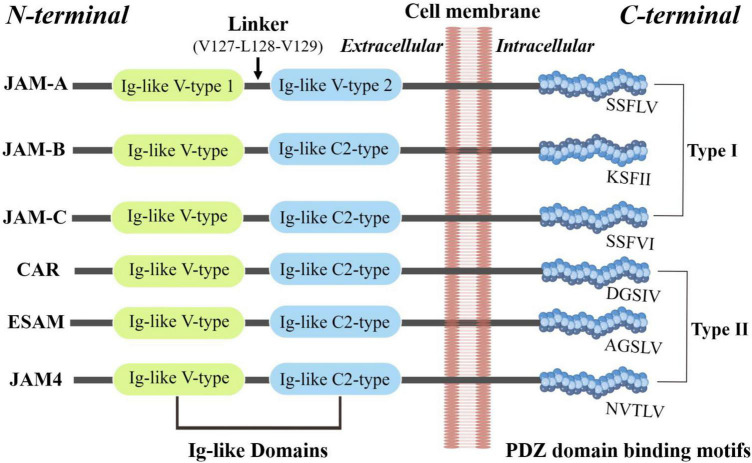
The basic structure of JAMs. JAM family proteins share common structural characteristics: a short *N*-terminal signal peptide, two extracellular Ig-like domains, a single transmembrane segment, and a short cytoplasmic tail with consensus phosphorylation sites and a *C*-terminal PDZ-binding motif (173).

JAM proteins are around 30–50 kDa in size. Kostrewa et al. and Prota et al. respectively, described the crystal structure of the recombinant extracellular part of mouse JAM (rsJAM) and human JAMs (hJAM) (3, 18). There is a linker region Val_127_–Leu_128_–Val_129_ between the N- and C-terminal domains and the extensive hydrogen bond network between the main chain atoms of the linker tri-peptide and both domains. The side chain of Leu_128_ is tightly packed in a hydrophobic pocket formed by the side chains of Gln_38_, Pro_40_, Thr_126_, Pro_159_, and Tyr_218_. Several proline residues (Pro_40_, Pro_130_, Pro_131_, Pro_159_, and Pro_160_) stabilize the main chain conformation around the linker (3). Interactions involving the membrane-distal Ig-like domain stabilize the dimer of mJAM1, similar to that observed in hJAM1. A dimer formed by two hJAM1 molecules is stabilized by extensive ionic and hydrophobic contacts between the N-terminal domains (18). These U-shaped dimers and salt bridges are then formed by a R(V,I,L)E motif (3), which is important in dimer formation and common among different JAMs, including rsJAM, hJAM, JAM-1, JAM-2, and JAM-3 (18). Dimerization and homophilic binding may contribute to both adhesive function and the junctional organization of JAMs (19). Figure 1 is a brief structural diagram of JAMs.

Mendoza et al. explored the extracellular domain of the JAM family and found each member has a unique tertiary structure despite having similar secondary structures, whose heterotypic interactions can be greatly favored compared to homotypic interactions (7).

### Interacting Proteins and Signaling Pathways

The physical and functional interactions with integrins contribute to the functions of JAMs to a great extent. The crosstalk between JAM-A and integrin αLβ2 (20–22), JAM-B and integrin α4β1 (23, 24), and JAM-C and integrin αLβ2 (LFA-1) (25, 26) mediates the transient interactions between leukocytes and endothelial cells. JAM-C expressed on platelets and integrin αMβ2 on leukocytes interact during inflammation (25). Meantime, JAM-integrin interactions in Cis also exert essential function, including JAM-A and JAM-C and integrin αVβ3 in endothelial cells (27–30), JAM-A and integrin αIIbβ3 in platelets (31–33), and JAM-L and integrin α4β1 in leukocytes (34). Accordingly, JAMs function substantially depending on physical and/or functional JAM-Integrin crosstalk (35).

JAMs build bridges between different cells through their interaction with PDZ domain-containing proteins, mainly relying on their COOH-terminal PDZ domain-binding motifs and adjacent domains (36–40). Besides, JAMs also consist of two tandem NH2-terminal, ectoplasmic Ig domains as well as single transmembrane spans (41). JAMs interact with a variety of cytoplasmic scaffolding proteins (42). Interaction of JAM with the tight junction (TJ) components of the PDZ domain-containing proteins ZO-1, cingulin, occluding, and AF-6 is the earliest evidence (36, 43). ZO-1 directly binds to the COOH termini of claudins and JAM by its PDZ1 and PDZ3 domains, respectively (44). In JAM-L cells, L fibroblasts overexpressed exogenous JAM, ZO-1 is concentrated at cell-cell contact sites (45). JAM-A resides in the correct localization of proteins involved in TJ formation, such as PAR-3, ZO-1, and MUPP1 (46). JAM-A interacts with Afadin and PDZ-GEF2 to activate Rap1A, regulating the levels of integrin β1 subunit and enhancing cell migration (47). JAM-A regulates epithelial permeability *via* association with ZO-2, Afadin, and PDZ-GEF1 to activate Rap2c and controls the contraction of the apical cytoskeleton (39). JAM-A is necessary for the development of polarity in cultured hepatic cells *via* its possible phosphorylation and recruitment of relevant PDZ proteins, linking to the apical domain (41). The nectin-afadin unit plays a role in the localization of JAM-1 at TJs (48), associated with the PAR-3-aPKC-PAR-6 complex. PAR-3 first binds to nectin-1 or -3 and is then transferred to JAM-1 during the organization of the junctional complex in the epithelial cells equipped with TJs (49). Both JAM2 and JAM1 possess an SFII or SFLV sequence on their intracellular tails predicted to interact with PDZ domains and therefore highly like to display similar binding activities (15, 50). JAM-A/C possibly affects TJ formation by influencing AF-6/afadin localization and/or function, which correlates with multiple structural components including nectins, ZO-1, and ponsin/SH3P12 (46, 51–53). Further research found ZO-1 and PAR-3 associated with JAM-2/-3 in a PDZ domain-dependent manner (54). The PAR3-JAM interaction is proposed to be reversible, but junctions will eventually form even in the presence of inhibitory PAR6 (55). In normal breast cells, JAM-A signaling *via* AF-6 and PDZ-GEF2 leads to a low level of β1-integrin-mediated cell migration (56). Besides, the RA175 forms a ternary complex with JAM-C *via* interaction with PAR-3, facilitating specialized adhesion structures (57).

The PSD95/dlg/ZO-1 (PDZ) domain of calcium/calmodulin-dependent serine protein kinase (CASK) and the putative PDZ-binding motif Phe-Leu-Val (COOH) in the cytoplasmic tail of JAMs is essential for association of the CASK and JAMs (44). The interaction that occurs between Grasp55 and JAM-C attributes to PDZ-mediated interaction with the C-terminal PDZ-binding motifs of protein cargos, playing a central role in stemness maintenance of hematopoietic and spermatogenic cells (58, 59). CASK co-localizes with both PMCA4b and JAM-A on the proximal principal piece, and acts as a common interacting partner of both to maintain Ca^2+^ homeostasis in sperm (60).

Hirabayashi et al. group has conducted several studies about the functional role of JAM4 and observed that JAM4 binds the scaffold protein MAGUK with inverted domain structure-1 (MAGI-1) but not to ZO-1 (6, 61–63). Besides, JAM4 directly binds the second PDZ domain of LNX1 through its carboxyl terminus (64). The newly discovered Mouse V-set and immunoglobulin domain containing 1 (VSIG1) interacts with Sertoli cells by heterophilic adhesion *via* its first Ig-like domain, critically depending on its binding to ZO-1 through the cytoplasmic domain (65). A PDZ domain-containing cytoplasmic protein, synaptojanin-2-binding protein (SYNJ2BP) expressed in human endothelial and epithelial cells was identified as a cytoplasmic binding partner of transmembrane and immunoglobulin domain-containing protein 1 (TMIGD1), another member of the Ig-superfamily (IgSF) similar to the JAM subfamily, affecting endothelial function (66, 67). In brain and muscle blood capillaries, JAM-related endothelial cell-selective adhesion molecule (ESAM) clearly co-localized with the three TJ markers occludin, claudin-5, and ZO-1 (68). Some hypothesized AS-associated JAMs-interacting proteins are shown in Table 1.

**TABLE 1:** Hypothesized atherosclerosis-associated JAMs-interacting proteins.

JAMs	Interaction molecules	Hypothesized function
JAM-A	Integrin αIIbβ3 (33) Integrin αLβ2 (20–22) Integrin αVβ3 (27–30) ZO-2, Afadin, PDZ-GEF1/2 (39, 47)	Mediate interactions of leukocytes and ECs; Increase platelet secretion and aggregation.
JAM-B	Integrin α4β1 (23, 24)	Mediate interactions of leukocytes and ECs.
JAM-C	Integrin αMβ2 (Mac-1) (25, 69) Integrin αLβ2 (LFA-1) (25, 26) Integrin αVβ3 (27–30)	Mediate interactions of leukocytes and ECs; Facilitate platelet phagocytosis.
JAM-L	Integrin α4β1 (34) ZO-1 (45), PAR-3 (54, 57).	Mediate interactions of leukocytes and ECs.
JAM4	MAGI-1 (6, 61–63)	Affect endothelial function.
TMIGD1	SYNJ2BP (66, 67)	Affect endothelial function.
ESAM	Occludin, claudin-5, ZO-1 (68)	Affect endothelial function.

### Main Physiological Functions

Based on topic IgSF structures and related structure research above, some ligands and receptors for JAMS have been discovered and assist the role of AMs in regulating cell motility, polarity, and proliferation in multiple cell types, including cancer cells, epithelial, endothelial, fibroblasts, leukocyte, and germ cells.

Various partner molecules and receptors bring about JAMs to exert their intracellular and intercellular functions within the body. Tight junctions (TJs) are structurally defined by electron microscopy and these epithelial intercellular junctions are located at the most apical region of cell-cell contacts (3, 70). Major JAMs members such as JAM-1, JAM-2, and JAM-3 are all located in the TJs of both epithelial and endothelial cells to preserve the structure of the junctions. These three JAMs molecules adhere to other tight junction proteins like PAR-3 and ZO-1. JAM-3 is unable to adhere to leukocytes in the manner as other JAMs do (54, 71). They assist TJs in exerting adhesive properties and stabilize homophilic cell-cell binding as a dual role in controlling paracellular permeability and in maintaining cell polarity (3).

But instead, JAM4 formulates TJs with MAGI-1 and plays a role in enhancing kidney cell branching and scattering (6, 61–63). Ligand-of-Numb protein X1 facilitates endocytosis of JAM4 and participates in transforming growth factor beta-induced redistribution of JAM4 in mammary epithelial cells (64). JAML exhibits its notable function in regulating cell migration *via* interacting with the tight junction protein coxsackie-adenovirus receptor (CAR), encompassing neutrophil [polymorphonuclear leukocytes (PMN)] transepithelial migration (72, 73), monocyte transendothelial migration (74), germ cells migration across the blood-testis barrier (75). The CAR group of proteins, composed of CAR, CLMP, BT-IgSF, and ESAM, might modulate the assembly or function of TJs (76). Because of the similar structure to the CAR group, how JAM groups operate in TJs deserves further attention.

It is noteworthy that the role JAMs play in cell migration is high-profile, covering endothelial cells, epithelial cells, germ cells, keratinocytes, tumor cells, hematopoietic stem, progenitor cells, and leukocytes such as lymphocytes, platelets, neutrophils, monocytes, and dendritic cells. The participation of JAMs in TJs supports a variety of biological processes both during development and in the adult organism, including developmental and physiological processes such as epithelial cell differentiation, hematopoiesis, germ cell development, and development of the nervous system, epithelial barrier formation, inflammation, angiogenesis, and hemostasis (35, 42, 77). As these functions exhibit potential links with AS, we focused on their functions directly related to AS in this review.

## Junctional Adhesion Molecules and Atherosclerosis

### Platelet Activation and Thrombosis

The adherence of platelet to inflamed endothelium is one of the most important initiated stages of plaque formation in blood vessels. Platelet activation also plays a pivotal role in atherothrombosis and related physiological processes involving clotting, fibrinolysis activation, and binding to the sub-endothelial matrix (78). Naik et al. first illustrated JAM-1, the F11-receptor (F11R), as a novel platelet membrane surface glycoprotein and a stimulatory monoclonal antibody, mAb F11, recognized JAM-1 and induced aggregation, adhesion, and potentiation in human platelets (79, 80). Kornecki team validated that sF11R (an F11R recombinant protein) inhibited this induction by two functional domains: the *N*-terminus domain and the 1st Ig-fold domain (81, 82). JAM-1 was proved to be selectively expressed at intercellular junctions between endothelial cells and platelets (68, 83, 84). There are type 1 and type 2 mRNAs of *JAM1*. Type 1 mRNAs exist in endothelial cells, platelets, leucocytes, and several cancer cells, while type 2 mRNAs are especially present in ECs, indicating their different functions in different cell types (85). Several amino acid residues including serine, threonine, and tyrosine within the external domain of JAM-1 can be phosphorylated (81, 82), and phosphorylation of Ser284 might engage platelet activation (86). JAM-1 increases platelet secretion and aggregation *via* promoting the assembly of the actin filament, relying on phosphoinositide-3 kinase activation and dimerization, phosphorylation of the 32 and 35 kDa forms, and combination with GPIIIa and CD9 (33).

The role of JAMs in platelet-endothelial adhesion in AS has also been extensively studied. Babinska et al. from the State University of New York devoted to studying the nexus between JAM-A (F11R) and AS, and observed high expression of JAM-A mRNA and protein in AS plaques from patients and *ApoE*^–/–^ mice (87). Plasma soluble JAM-A (sJAM-A) independently correlated with the severity of coronary arterial disease (CAD) defined by angiographic score and plasma levels of Tumor necrosis factor-α (TNF-α) (88). More interestingly, transmembrane-JAM-A and sJAM-A from platelet act as homophilic interaction partners to exacerbate thrombotic and thrombo-inflammatory interactions between platelet and monocyte (89). JAM-A expressed and adhered to cultured cytokine-inflamed ECs from human aortic and venous vessels (90), which was inhibited by treatment with actinomycin, parthenolide, or AG-480, similar to the result caused by *Jam-A* siRNAs, which related to NF-kappaB and JAK/STAT pathways (91). Crosslinking of platelet F11R/JAM-A with the FcγRII by monoclonal antibody F11 (M.Ab.F11) caused platelet aggregation, resulting from phosphoinositide-3 kinase-triggered actin filament assembly (33, 90). JAM-A mediates platelet adhesion and spread through filopodial extensions and lammelipodia development (33). Alternatively, supersensitivity of platelets to natural agonists thrombin and collagen with JAM-A stimulation is independent of the Fc gammaRII (33). Babinska et al. group developed F11R/JAM-A antagonistic [peptide 2HN-(dK)-SVT-(dR)-EDTGTYTC-CONH2, F11R peptide 4D] as a potential anti-atherosclerotic and/or anti-thrombotic therapeutic drug and confirmed that the F11R peptide 4D inhibited M.Ab.F11-induced platelet aggregation and cytokine-inflamed platelets adhesion to ECs, remarkably, reduced atherosclerotic plaque formation and inhibited platelet adhesion to the cytokine-inflamed arterial endothelium in *ApoE^–/–^* mice (92–94). Naik et al. reported thrombotic function of platelets was enhanced in *Jam-A^–/–^* mice *in vivo* and JAM-A suppressed integrin αIIbβ3 outside-in signaling to limit platelet accumulation and prevent premature platelet activation (31, 32). When stimulated by an agonist, the dephosphorylation of JAM-A on the tyrosine residue allowed the dissociation of JAM-A-recruited Csk from the integrin-c-Src complex and thus facilitated outside-in signaling (31, 32). Koenen group generated platelet-specific (tr) *Jam-A*-deficiency in *ApoE^–/–^* mice (*trJam-A^–/–^ ApoE^–/–^*) and observed gain-of-function in platelets with more αIIbβ3 signaling-related proinflammatory effects, increased aortic plaque formation, and accelerated neointima formation in earlier stages after vascular injury (95, 96). JAM-A is associated with hypertension in humans, and JAM-A protein is upregulated in the brainstem microvasculature and brain endothelium in hypertensive rats models, possibly activated by AT (1) receptor-mediated signaling (97, 98). In rats injected with ADP-activated platelets, JAM-A and CD41 co-localized in the microvessels (98).

JAM-C was expressed mostly on human platelets in mono- and dimeric forms and endothelial JAM-C was supposed to function in synergy with platelets JAM-C in the development of CAD (99). Direct interaction of human platelet JAM-C with myelomonocytic U937 cells, neutrophils, and dendritic cell β2-integrin Mac-1 (integrin αMβ2, CD11b/CD18) was corroborated to facilitate DC activation and platelet phagocytosis, aggravating the progression of atherosclerotic plaque (25, 69). sJAM-C markedly reduced the adhesion of DCs to platelets, purified JAM-3 blocked the platelet-neutrophil interaction and anti-JAM-C decreased platelet activation, platelet-neutrophil aggregation, and platelet macroaggregates, but greatly increased neutrophil degranulation (25, 69, 100). Antibodies against JAM-C thereby served as a prospective antibody to prevent atherothrombosis and AS.

In summary, the expression of JAM-A and JAM-C in platelets mediate the aggregation and adhesion of platelets and potentiate their role in atherothrombosis and AS. Soluble JAM-A correlates with the severity of CAD. Antibodies against JAM-A and JAM-C are promising for anti-atherosclerosis drug development.

### Vascular Inflammation and Leukocyte Infiltration

Vascular inflammation is critically important in AS, one of the characteristics is the recruitment of leukocytes into the inflamed artery wall (101). Monocytes and the derived macrophages contribute to all stages of AS, including the recruitment into the intima, secretion of inflammatory cytokines, lipid accumulation, plaque progression, maturity, and break (102). JAMs play important roles in inflammation in various diseases. More serious steatohepatitis emerged in *Jam-A^–/–^* mice fed with a diet high in saturated fat, fructose, and cholesterol (HFCD) (103). Tyrosine phosphorylation of JAM-A (p-Y280) caused loss of epithelial barrier function during intestinal inflammation (104). JAM-A stably expressed on iPSC-cardiomyocytes (iPSC-CM) contributes to iPSC-CM inflammation (105). JAM-A is also necessary for peripheral mononuclear cells (PMN) infiltration into the heart upon ischemia-reperfusion injury (106). For vascular inflammation, Kiessling depicts JAM-A as the indicator of inflammatory arterial areas exposed to acutely alternated flow, as determined by molecular ultrasound imaging (107). TNF stimulates the disassembly of JAM-A from the TJs and subsequent redistribution as well as dispersal on the endothelial cell surface, which was reduced by fibronectin (108). Nonetheless, the loss and/or redistribution of JAM-A induced by the combined treatment of TNF-α and IFN-γ in ECs scarcely regulated leukocyte adhesion or transmigration (109). In some cases, endothelial JAM-A seems to not contribute to leukocyte adhesion or transcellular migration (110, 111). Shedding of soluble JAM-A (sJAM-A) on inflamed vascular endothelium mediated predominantly *via* ADAM17 and slightly by ADAM10 may signal vascular inflammation (112). ADAM17 surface expression and JAM-A cleavage are increased by flow cultivation (113), which results in impaired endothelial wall shear stress (WSS) mechanosensing (114). *Jam-C^–/–^* mice and mice treated with anti-JAM-C antibody present a significant reduction in intranodal homeostatic chemokine secretion, which in turn suppressed naive T cell exit from lymph nodes (115). Likewise, JAM-C antibodies substantially curtailed systemic and lung inflammatory cytokines and chemokine as well as pro-inflammatory aged neutrophils (116). Imaginably, the cyclic nitroxide 4-MethoxyTEMPO treatment minimized inflammatory cell recruitment into human aortic EC *via* JAM-C blockade (117).

As a biomarker of cell adhesion, JAM-A was independently associated with the endpoint of stable patients with chronic heart failure (CHF) (118). Also, sJAM-A secreted from cardiac progenitor cells attenuated neutrophil infiltration after myocardial infarction. Accumulating evidence has indicated that JAM-A accelerates the formation of AS lesions in mice. Oxidized low-density lipoprotein (ox-LDL) up-regulates *Jam-A* mRNA expression in human macrophages and human umbilical vein endothelial cells (HUVECs), stimulates the redistribution of JAM-A in ECs, and subsequently increases the transmigration of monocytes, which could be counteracted by statins (119–122). Christian Weber’s group conducted long-term studies on the role of JAM-A in AS. In *ApoE^–/–^* mice fed with an atherogenic diet, endothelial JAM-A expression was upregulated, while JAM-A deficient mice displayed decreased neointima hyperplasia, reduced macrophage content, and attenuated monocyte arrest and transmigration in carotid arteries (123, 124). Atherosclerotic lesion formation in *ApoE^–/–^* mice was reduced by endothelial JAM-A deficiency with limited monocyte recruitment into the arterial wall but was aggregated by myeloid JAM-A deficiency with impaired monocyte de-adhesion (122). Compared to *ApoE^–/–^Ldlr^–/–^* mice, *ApoE*^h/h^*Ldlr^–/–^* mice showed decreased endothelial JAM-A expression in the aortic arch (125). sJAM-A effectively blocked the recruitment of monocytes to atherosclerotic endothelium (123). Mechanically, the treatment of JAM-A siRNA in aortic endothelial cells inhibited the transmigration of monocytes (121). JAM-A was repressed by microRNA (miR)-145, which was upregulated under atheroprotective laminar flow (122). miR-145-rich exosomes inhibited the development of AS by downregulating JAM-A (126). JAM-A was suppressed by miR-156a in human aortic endothelial cells, which in turn depressed inflammatory monocyte adhesion induced by cytokines (127). Bilberry anthocyanin-rich extract is observed to attenuate AS development in *ApoE^–/–^* mice by downregulating the expression of *Jam-A* (128). Ginkgolide B almost abolished the upregulation of JAM-A expression in HUVECs induced by ox-LDL (119). In the *Ldlr^–/–^* mice fed with fish oil, the atherosclerotic lesions were diminished, which was accompanied by reduced circulating endothelial cell JAM-A expression (129).

An antibody against JAM-C reduced monocyte accumulation at vascular inflammation sites by increasing reverse transmigration instead of reducing transmigration (130). Exosomal miR-146a-5p bound to the 3’-untranslated region of *JAM-C* mRNA to curb monocyte transendothelial migration (131). In *ApoE^–/–^* mice, JAM-C expression was upregulated as spontaneous early lesions developed (132). Anti-JAM-C antibody caused increased reverse transendothelial migration (rTEM) of monocyte-derived cells as well as decreased neointima hyperplasia and neointima macrophage induced by wire injury of carotid arteries and atherogenic diet (133, 134). oxLDL upregulated JAM-C expression and induced disorganization of JAM-C localization on ECs (132). Blockage of JAM-C diminished monocyte arrest and adhesion to carotid artery smooth muscle cells (SMCs) under flow conditions (133). Unexpectedly, overexpression or gene silencing of JAM-C in human ECs under flow conditions showed similar higher rates of monocyte rTEM (134). JAML was induced during the differentiation of myeloid leukemia cells and promoted integrin-mediated adhesion of leukocytes to ECs (8, 34). JAML expression also correlated with the adhesion and TEM of monocytes (74). JAML was highly expressed in atherosclerotic plaques of *ApoE^–/–^* mice and AS patients as well as macrophages exposed to oxLDL (135). Silencing JAML expression attenuated the formation of atherosclerotic lesions and promoted plaque stability, possibly resulting from decreased expression of inflammatory cytokines (135, 136).

Overall, the expression of JAM-A, JAM-C, and JAML in ECs was increased in an atherosclerotic environment. These molecules play important roles in the recruitment and TEM of leucocytes into the intima. Related mediators and pathways remain to be investigated in deep. Soluble JAM-A and the antibody against JAM-C blocked the recruitment of monocytes to inflamed atherosclerotic endothelium. Silencing the expression of *Jam-A* and *JamL* attenuated AS.

### Endothelial Barrier and Angiogenesis

Damage to the endothelial barrier and angiogenesis are essential events for plaque formation. The changes in the expression of related angiogenic factors, blood flow, nutrients, O_2_, and other events caused by neovascularization may take part in plaque progression, remodeling, destabilization, and thromboembolic events (137). JAM-A mediates human CD34^+^ progenitor cells differentiating into endothelial progenitor cells through interaction with LFA-1 (138). In the rat cortical cold injury model, JAM-A co-localized with occludin at TJs in the lesion vessels with blood-brain barrier (BBB) breakdown and expressed decreasingly at 12 h only (139). Although human brain EC (HBMEC) released sJAM-A into culture supernatants with non-inducibility, which favors protecting brain EC from inflammatory stimuli, sJAM-A was still speculated unsuitable as a biomarker of BBB breakdown (140). Mechanism study showed that JAM-A promoted CCAAT/enhancer-binding protein-α (C/EBP-α) expression by suppressing β-catenin transcriptional activity and by activating exchange protein directly activated by cAMP (EPAC), thereby increasing transcription of downstream target Claudin-5 to decrease endothelial permeability and enhance vascular barrier function (141). Moreover, Insulin-like growth factor-1 (IGF-1) was reported to upregulate JAM-A expression and further protect endothelial barrier function in human aortic endothelial cells (142). Similarly, Tongxinluo (a special formula of Chinese traditional medicines) reversed the endothelial barrier breakdown with enhanced expression of JAM-A (143, 144). JAM-A was proposed to disassemble the junctions caused by TNF (108). Strikingly, the levels of plasma sJAM-A and EC-expressed JAM-A protein were reduced by the tumor inducers Tβ4 and TGF-β1, and the F11R/JAM-A antagonistic peptide 4D (P4D) showed a prospective barrier-protecting effect (145). The junction structure reorganization for JAM-A changed under low laminar flow conditions (146). Of note, JAM-A rearranged from interendothelial TJs to the luminal surface of blood vessels under acute blood flow variations, which implied JAM-A as a marker of acute endothelial activation and dysfunction (147). Melatonin upregulated the expression of tight junction proteins to maintain the rat inner blood-retinal barrier (iBRB) integrity, such as ZO-1, Occludin, JAM-A, and Claudin-5 (148). In mouse retinal vascular, JAM-C deficiency increased the spreading of fibronectin and consequently enhanced endothelial cell sprouting and vessel normalization *in vitro*, dependent on β1-integrin and the small GTPase Rap1 activation (149). Consistently, VEGF or PDGF-C induced JAM-C translocating from cytoplasm to the cytomembrane to maintain the normal function of the human iBRB, while increased serum sJAM-C was identified as a potential marker of wet age-related macular degeneration (wAMD) (150). Besides, JAM-A, JAM-B, and especially JAM-C have readily been detected in liver sinusoidal endothelial cells (LSECs), as a part of special mixed-type intercellular junctions (151).

JAM-A is expressed prominently in embryonic vasculature and on the surface of hematopoietic precursors and has the potential to mark isolate long-term reconstituting HSC (LTR-HSC) (152, 153). JAM-A blocking mAb inhibited angiogenesis *in vitro*, in the embryo, and *in vivo* (154). FGF-2-induced microvessel sprouting failed in *Jam-A^–/–^* mice (155). JAM-A cleavage-mediated abnormal arterial remodeling in aging was also regulated by ADAM17 (114). Transfection with *Jam-b* siRNA promoted vessel sprouting, and intraperitoneal administration of anti-JAM-B antibody increased tumor blood vessel density, as unanimously observed in JAM-B-heterozygous mice (156). On the contrary, anti-JAM-C monoclonal antibody decreased vessel development from aortic rings *in vitro* and angiogenesis in the model of hypoxia-induced retinal neovascularization without pathological side effects *in vivo* (157). Unexpectedly, disrupting the interactions of JAM-B and JAM-C cytoplasmic tails and PAR3 with antibodies, siRNA, or dominant-negative mutants fully interferes with EC lumen formation and tubulogenesis (158).

Even though JAMs were proved to be functional in endothelial barrier and angiogenesis, there is still insufficient evidence supporting their key roles in atherosclerotic vascular endothelial barrier and angiogenesis in AS. The potential is worthy of further exploration.

### Shear Stress and Cell Motility

Of equal concern for the development of AS and complications is wall shear stress (WSS). Chronically low oscillating WSS is most susceptible to causing local AS. However, stenosis-induced high WSS pushes plaque rupture (159, 160). As several of above mentioned AS-related events such as vascular inflammation, disruption of endothelial barrier, and angiogenesis are closed related to flow-induced shear stress, we made a separate summary in this section.

Without shear stress, *Jam-A* knock-out accelerated cell motility by enhanced directional persistence. Under shear stress, *Jam-A* knock-out escalated protrusion extension at the flow direction and elevated downstream cellular displacement (161). JAM-A shedding could be increased with ADAM17 maturation. Responded to flow exposure, the shedding of JAM-A increased but the *Jam-A* mRNA expression was retained (113). Noteworthy, ADAM17-activation and JAM-A/F11R cleavage impaired endothelial WSS mechanosensing (162).

Under high shear stress, JAM-A was expressed on human CD34^+^ progenitor cells and significantly decreased adhesion over immobilized platelets or inflammatory endothelium (138). The Mian Long group found that JAM-A and JAM-C were the ligands of Mac-1 when mediating PMN adhesion. Under high shear stress, LFA-1/Mac-1-JAM-C bonds hastened PMN crawling (163). Under shear flow at the physiological level, the high bond strength between LFA-1 and JAM-A raised a strong Ca^2+^ response in adherent PMNs especially (164). Rolling and sticking interactions of immobilized JAM-B protein with human T lymphocytes were barely observed at a high shear stress (1.0 dyn/cm^2^) but readily observed at a lower shear stress (0.3 dyn/cm^2^) (24).

Taken together, it is necessary to consider the flow-induced shear stress when discussing the roles of JAMs in AS, especially related to endothelial inflammation and leucocytes motility. More precise conditions of shear stress deserved to be explored.

### Vascular Intercellular Interactions

Essentially, AS is caused by a series of cellular interactions. Multiple scRNA-seq data of plaques from mice and humans demonstrated the development of atherosclerosis resulting from the combined action of various types of cells (165, 166). As described above, monocytes TEM plays a vital role in the development of AS. At the same time, some additional research revealed the importance of other interactions. T cells are the second key leukocyte population in atherosclerotic lesions (167). JAM-A functioned in the interactions between CD4^+^ T cells and DC, possibly concerning vascular inflammation (168). sJAM-A can diminish the chemotaxis of activated T cells triggered by stromal cell-derived factor (SDF)-1α-transendothelial (123). Moreover, JAM-A expressed by human CD34^+^ cells regulated the interactions between platelets and endothelial cells to mediate the adhesion of platelet to inflammatory endothelium (138). Neutrophils play a role in vascular inflammation and plaque formation as well (169). JAM-A was also supposed to be an endothelial receptor of neutrophil transmigration (170). The JAM-C mediated neutrophil transmigration is dependent on Mac-1 (171). As a subsequent event in plaque formation, the proliferation and migration of inflamed smooth muscle cells seem to be inseparable from JAM-A (172).

## Conclusion and Prospective

**FIGURE 2:**
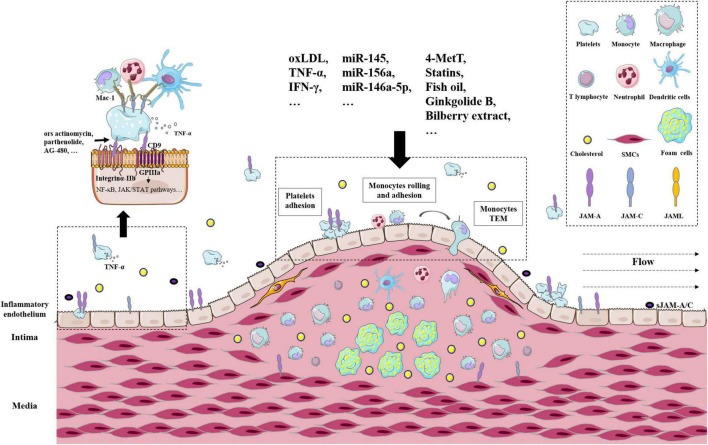
The roles of JAMs in atherosclerosis arteries. Representative receptors, pathways, and regulators of JAMs in atherosclerotic plaques. Related cell types include platelets, leucocytes, endothelial cells, and vascular smooth muscle cells.

Taken together, a large body of evidence supports the crucial role of JAMs in AS. JAM-A and JAM-C were typically highly expressed in cellular components of atherosclerotic plaques from patients and *ApoE^–/–^* mice, including platelets, leucocytes, endothelial cells, and vascular smooth muscle cells (25, 69, 87). Representative receptors, pathways, and regulators were delineated in Figure 2. There are many issues needed to be studied. JAMs are expressed in different types of cells in AS plaque, but the similarities and differences between these JAMs are ambiguous. What are the downstream effects of different JAMs in different cells? Do JAMs mediate the interactions between these cells? If they do, how do they achieve that? How do environmental factors change those functions? The above questions still need to be answered in future studies.

Various models of animals and reagents have been developed in the functional study of JAMs. *Jam-A^–/–^* mice, *Jam-C^–/–^* mice, *Jam-B*-heterozygous mice (156), platelet-specific (tr) *Jam-A*-deficiency mice (*trJam-A^–/–^*) (95, 96), and their hybrid mice crossed with *ApoE^–/–^* mice have been used to illustrate the related mechanisms. sJAM-A and sJAM-C have been purified to demonstrate their physiological functions. Antibodies against JAM-A, JAM-B, and JAM-C have been also prepared and validated.

The value of JAMs in AS is reflected as potential anti-atherosclerotic and/or anti-thrombotic therapeutic targets. F11R/JAM-A antagonistic (F11Rpeptide 4D) developed by the Babinska et al. group (92–94) and antibodies against JAM-C demonstrated the potential to prevent the development of atherothrombosis and AS. The plasma level of JAM-A correlates with the severity of CAD, which indicates a potential biomarker for the disease (88). More data are awaited to show their value in clinical application.

## Author Contributions

JW conceptualized and wrote the manuscript. XPC checked and revised the manuscript. Both authors contributed to the article and approved the submitted version.

## Conflict of Interest

The authors declare that the research was conducted in the absence of any commercial or financial relationships that could be construed as a potential conflict of interest.

## Publisher’s Note

All claims expressed in this article are solely those of the authors and do not necessarily represent those of their affiliated organizations, or those of the publisher, the editors and the reviewers. Any product that may be evaluated in this article, or claim that may be made by its manufacturer, is not guaranteed or endorsed by the publisher.
